# Characterization of β-glucosidase of *Lactobacillus plantarum* FSO1 and *Candida pelliculosa* L18 isolated from traditional fermented green olive

**DOI:** 10.1186/s43141-021-00213-3

**Published:** 2021-08-09

**Authors:** Yahya Rokni, Houssam Abouloifa, Reda Bellaouchi, Ismail Hasnaoui, Sara Gaamouche, Zahra Lamzira, Riadh B. E. N. Salah, Ennouamane Saalaoui, Nabil Ghabbour, Abdeslam Asehraou

**Affiliations:** 1 Laboratory of Bioresources, Biotechnology, Ethnopharmacology and Health, Faculty of Sciences, Mohammed Premier University, BP 717, Oujda, Morocco; 2 Laboratory of Microorganisms and Biomolecules, Centre of Biotechnology of Sfax, BP: 1177, 3018 Sfax, Tunisia

**Keywords:** Olive, Oleuropein, β-Glucosidase, *Lactobacillus plantarum*, *Candida pelliculosa*

## Abstract

**Background:**

Oleuropein, the main bitter phenolic glucoside responsible for green olive bitterness, may be degraded by the β-glucosidase enzyme to release glucose and phenolic compounds.

**Results:**

*Lactobacillus plantarum* FSO1 and *Candida pelliculosa* L18 strains, isolated from natural fermented green olives, were tested for their β-glucosidase production and activity at different initial pH, NaCl concentrations, and temperature. The results showed that strains produced extracellular and induced β-glucosidase, with a molecular weight of 60 kD. The strains demonstrated their biodegradation capacity of oleuropein, associated with the accumulation of hydroxytyrosol and other phenolic compounds, resulting in antioxidant activity values significantly higher than that of ascorbic acid. The highest production value of β-glucosidase was 0.91 U/ml obtained at pH 5 and pH 6, respectively for *L. plantarum* FSO1 and *C. pelliculosa* L18. The increase of NaCl concentration, from 0 to 10% (w/v), inhibited the production of β-glucosidase for both strains. However, the β-glucosidase was activated with an increase of NaCl concentration, with a maximum activity obtained at 8% NaCl (w/v). The enzyme activity was optimal at pH 5 for both strains, while the optimum temperature was 45 °C for *L. plantarum* FSO1 and 35 °C for *C. pelliculosa* L18.

**Conclusions:**

*L. plantarum* FSO1 and *C. pelliculosa* L18 strains showed their ability to produce an extracellular and induced β-glucosidase enzyme with promising traits for application in the biological processing of table olives.

## Background

Oleuropein is the main natural polyphenol glucoside responsible for olive bitterness [1]. The olive debittering process, widely practiced in the table olive industry, is based on the lye solution (NaOH) treatment of fruits. However, this chemical process is associated with drawbacks on the consumer’s health by reducing nutrients in fermented olives, and on the environment by releasing toxic wastewaters rich in sodium hydroxide [2]. The biological debittering of olives, based on starters with a specific enzyme, can contribute to the sustainable development of this bio-resource.

Oleuropein, an *O-*glycosylated compound composed of d-glucose β (1-4) linked to aglycone, is reported to be hydrolyzable by the β-glucosidase enzyme, releasing d-glucose and aglycone [1, 3]. The latter is subsequently hydrolyzed with esterase to simpler phenolic compounds, namely hydroxytyrosol and elenolic acid [1, 4]. This hydrolysis plays an important role in enhancing the lactic fermentation process of olives by, providing glucose as a carbon source for lactic acid bacteria [5], and increasing the nutraceutical property of fermented olives with hydroxytyrosol accumulation [6].

Based on the Carbohydrate-Active Enzyme database (http://www.cazy.org, CAZy) [7], the β-glucosidase enzymes are of a class of glycoside hydrolases (EC 3.2.1-) [7]. These enzymes catalyze the hydrolysis of *O-*glycosidic bonds between two or more carbohydrates or between a carbohydrate and a non-carbohydrate moiety (aglycone), leading to the release of carbohydrate and aglycone [7–9]. The β-glucosidase enzymes are widespread in microorganisms, and their genes of production can differ in strains of the same species [9].

Several studies reported the capacity of *Lactobacillus* strains (*L. plantarum*, *L. casei*, and *L. brevis*) [6, 10–12] and yeasts [13, 14] to produce the β-glucosidase enzyme. In previous work, we reported high in vitro β-glucosidase activity of some *Lactobacillus* strains, releasing glucose and hydroxytyrosol from oleuropein [15]. However, the β-glucosidase activity of *L. plantarum* strain obtained was lower in the olive brine environment and led to the production of a fermented green olive with some bitterness [16]. The in vitro biodegradation of oleuropein by *L. plantarum* was demonstrated to be dependent on the stress factors (pH, NaCl, and glucose) [6]. The conditions of production and activity of β-glucosidase of *Lactobacillus* and yeast strains, particularly in olive brine conditions, are not well highlighted. Further studies elucidating this biodegradation process, could improve the biological debittering process of green olives, by using selected microorganisms and/or their β-glucosidase. The main objective of this work is to study the production and activity of β-glucosidase of *L. plantarum* FSO1 and *Candida pelliculosa* L18 strains, isolated from traditional fermented green olives, in presence of stress factors close to olive brine environment.

## Methods

### Microorganisms studied

The microbial strains used in this work were *Lactobacillus plantarum* FSO1 and *Candida pelliculosa* L18, isolated in our laboratory from traditional fermented green olive brine [17]. In this traditional process, non-debittered green olives of Moroccan Picholine variety were directly brined and led to undergo a natural fermentation process, allowing the transformation of olives into an edible product.

### Chemicals and reagents

All the chemicals used in this work were purchased from Sigma-Aldrich. The solvents (methanol, toluene, and acetic acid) were of analytical grade. The commercial enzyme beta-glucosidase (EC.3.2.1.21) was purchased from Sigma-Aldrich.

### Detection of β-glucosidase enzyme

#### API-ZYM gallery

The enzymatic profile of the strains of *L. plantarum* FSO1 and *C. pelliculosa* L18 was determined using the API-ZYM gallery (BioMerieux, France). This gallery (ref 25200) is a simplified semi-quantitative evaluation technique for 19 enzymes [18]. It was used to highlight the enzymatic profile of the strains, including β-glucosidase and esterase.

The 20 wells of the gallery were inoculated with 65 μl of overnight cultures of *L. plantarum* FSO1 and *C. pelliculosa* L18, obtained on De Man Rogosa and Sharpe (MRS) and Yeast Extract Glucose (YEG) broth, respectively. The microbial suspensions were standardized to 5-6 Mac Farland. After inoculation, the gallery was incubated at 28 °C for 5 h, then a drop of each reagent ZYM A (Ref 70494) and ZYM B (Ref 70493) was added to each well to develop the stain revealing enzyme activity. After 5 min, a numerical value from 0 to 5 was assigned based on the evaluation of the color developed in each enzyme reaction, using the color card provided by the manufacturer [19, 20].

#### Esculin-based medium

The esculin-based medium was used to detect the β-glucosidase activity in *L. plantarum* FSO1 and *C. pelliculosa* L18, according to the method of [21]. Briefly, the medium composed of casein peptone 8 g/L, esculin sesquihydrate 1 g/L, ferric ammonium citrate 1 g/L, agar 17 g/L, pH 7.4, is autoclaved and then poured in Petri dishes. Overnight cultures (60 μl) of the strains (FSO1 and L18) were drop inoculated on the medium and then incubated at 30 °C for 48 h. Sixty microliters of β-glucosidase (2mg/ml) and distilled water were used as positive and negative controls, respectively. After incubation of the culture assays, made in triplicate, a positive β-glucosidase activity is indicated by a black precipitate around colonies, due to the hydrolysis of esculin to glucose and esculetin. The esculetin reacts with iron III ions to form a black precipitate.

### Induction and localization of β-glucosidase activity

The strains (*L. plantarum* FSO1 and *C. pelliculosa* L18) were tested for the induction and localization (extracellular, intracellular, or membrane) of β-glucosidase activity. For this, overnight cultures (5 μL) of the strains (FSO1 and L18) were inoculated in 5 mL of normal MRS and modified MRS (MRSm), containing 1% (w/v) of oleuropein (Extrasynthese, Genay, France) as a sole carbon source. After incubation at 30 °C for 7 days, the obtained cultures were centrifuged at 12000 g for 12 min at 4 °C (Hermle Labnet Z216MK). The supernatant (supernatant 1: *E*) obtained was separated from the pellet (cell cream). The cell cream was then resuspended in 0.1 M phosphate buffer at pH 5, and then the cells were lysed by sonication at 4 °C for 45 min in an ultrasonic bath (Bandelin Sonorex Digitec) with continuous and pulsed modes of 5 s ON/5 s OFF according to the method of Pchelintsev, Adams [22]. The lysed cells were centrifuged, at 12000×g for 12 min at 4 °C, to separate the intracellular and the membrane fractions. After these operations three fractions were obtained for each strain, the extracellular (supernatant 1: *E*), the intracellular (supernatant 2: I), and the membrane pellet (cell wall suspension: *M*) fractions. These fractions (*E*, *I*, and *M*) were used to measure the enzymatic activity.

### Sodium dodecyl sulfate-polyacrylamide gel electrophoresis

The fraction E, obtained with oleuropein (1%) as a sole carbon source, was added with (NH_4_)_2_ SO_4_ 80% (w/v) to precipitate proteins. After overnight precipitation, the precipitates formed were collected by centrifugation at 10000 × g for 20 min at 4 °C, and the pellets obtained were dissolved in 20 mM sodium acetate buffer pH 5.0. The sodium dodecyl sulfate (SDS) gel was prepared with 0.1% SDS in 15% separating gels and 5.0% stacking gels (final gel concentration). Tris-glycine buffer pH 8.3 containing 0.1% SDS was used as the electrode buffer. Discontinuous SDS-PAGE in reducing conditions was performed according to the procedure of Laemmli [23]. Samples were treated with Laemmli buffer and boiled for 10 min at 95 °C before application to the gel. Electrophoresis was run from cathode to anode at 130 V, 30 mA for 40 min at room temperature in a Mini-Gel Electrophoresis Unit (Bio-Rad). The following proteins were used for calibration: lysozyme (14.6 kDa), esterase (28 kDa), β-glucosidase (60 kDa), bovine serum albumin (66 kDa), glucose oxidase (160 kDa). After running electrophoresis, proteins in the gel were visualized by staining with silver nitrate, according to the method of Wray et al. [24].

### Determination of β-glucosidase activity

The β-glucosidase activity of the fractions (*E*, *I*, and *M*) obtained from the strains (FSO1 and L18) was determined according to the method of Norkrans [25], by measuring the hydrolysis of para-nitrophenyl-β-d-glucopyranoside (p-NPG) (Merck) used as substrate. The reaction mixture contained 0.9 mL of p-NPG (5 mM) in a 50 mM citrate buffer (pH 4.8) and 0.1 mL of the enzymatic fraction (*E*, *I*, or *M*), obtained in presence of oleuropein (1%) as a sole carbon source. The reaction was carried out at 50 °C for 10 min and then stopped by the addition of 2 mL of Na_2_CO_3_ (1M) and 10 mL of distilled water. The amount of para-nitrophenol (p-NP) released was determined by measuring its absorbance at 405 nm. One unit of enzymatic activity (U) was defined as the amount of enzyme which produced 1 μmol of p-NP (para-nitrophenol) per min under the conditions of the experiment. A calibration curve was prepared using p-NP. All the tests were carried out in triplicate.

### Antioxidant activity

The antioxidant activity of the extracellular fraction, of the strains FSO1 and L18, and their combination (FSO1/L18) obtained on MRSm after 7 days of incubation at 30 °C, was evaluated by measuring the DPPH (2,2-diphenyl-1-picrylhydrazyl) free radical scavenging effect, according to the method of [26], with some modifications. Briefly, 50 μl of the sample (extracellular fraction) was added to 1.95ml of freshly prepared DPPH (25 mg/l in methanol). After 40 min of incubation in dark at room temperature, the absorbance was measured at 517 nm against a blank, consisting of 200 μl of DPPH solution and 100 μl of methanol. Ascorbic acid (0-100 μg/ml) was used as control. The antioxidant activity (AA, %) was calculated using the following equation: AA (%) = ((*A*_0_ − *A*_1_)/A_0_) × 100, where *A*_0_ was the absorbance of the blank, and *A*_1_ was the absorbance of the sample of extracts and standards. Three replicates were taken for each assay.

### β-Glucosidase production conditions

#### Effect of incubation time

The in vitro monitoring of β-glucosidase production was determined by measuring the hydrolysis rate of p-NPG, to β-d-glucose and para-nitrophenol, by β-glucosidase of the fractions *E* of the strains FSO1, L18 and their combination (FSO1/L18). For this, 10 μL of overnight cultures of each strain (5 μL each of each strain when combined, FSO1/L18) were inoculated in 10 mL of modified MRSm broth, containing oleuropein (1%, w/v) as a sole carbon source. The cultures, made in triplicate, were incubated at 30 °C from one day to 9 days. Samples were harvested from the cultures and analyzed for their β-glucosidase activity and biomass content. The β-glucosidase activity was measured as described above. The biomass was measured by enumeration of *L. plantarum* FSO1 on MRS agar and *C. pelliculosa* L18 on Potato Dextrose Agar (PDA). Cycloheximide (0.01%, w/v) was added to MRS to prevent the growth of yeast, while gentamicin (4%, w/v) was added to PDA to prevent the growth *of L. plantarum*.

#### Effect of pH

The effect of pH on the production of β-glucosidase, by the strains FSO1 and L18 and their combination, was determined by measuring the β-glucosidase activity of their culture obtained on MRSm broth adjusted to different initial pHs (4, 5, and 6), and inoculated with 10 μl of overnight cultures of the strains (FSO1, L18, and FSO1/L18), as described above. After 7 days of incubation at 30 °C, the β-glucosidase activity was measured on the extracellular fraction, using the same protocol described above. Two controls were used in this experiment, citrate buffer (50 mM at pH 4.8) as negative control and commercial β-glucosidase (EC 3.2.1.21, Sigma-Aldrich) as a positive control. All the experiments were performed in triplicate.

#### Effect of NaCl

The effect of NaCl on the production of β-glucosidase by the strains studied (FSO1, L18, and FSO1/L18) was determined by measuring the β-glucosidase activity of their cultures obtained on MRSm broth adjusted to different NaCl concentrations (0, 2, 4, 6, 8, and 10%, w/v), and inoculated with overnight cultures as described above. After 7 days of incubation at 30 °C, the β-glucosidase activity was measured on the extracellular fraction using the same protocol described above. Two controls were used in this experiment, citrate buffer (50 mM at pH 4.8) as negative control and commercial β-glucosidase (EC 3.2.1.21) as a positive control. All the experiments were performed in triplicate.

#### Confirmation of enzymatic activity by TLC

To confirm the biodegradation of oleuropein by the strains, cultures of *L. plantarum* FSO1, *C. pelliculosa* L18, and their combination (FSO1/L18) were carried out on MRSm broth. After 7 days of incubation at 30 °C, the phenolic compounds of the microbial cultures were extracted three times with ethyl acetate (8:2, v/v). After decantation, the organic phase was harvested and left in the dark for 30 min in the presence of disodium sulfate, and then dry evaporated at 50 °C. The residue obtained was dissolved in 1 mL of methanol. The phenolic extracts obtained were dissolved in 1 ml of methanol and then subjected to Silica-gel thin layer chromatography (TLC) (Silica gel/TLC-cards, Fluka-60778) to separate the phenolic compounds resulting from the degradation of oleuropein. The phenolic extracts and the standards (oleuropein and hydroxytyrosol), prepared in methanol, were deposited on the TLC cards using a glass capillary. The eluent used was composed of toluene/methanol/acetic acid (15/5/0.5) and (15/5/1) [27]. The observation was realized, when the solvent front reached the upper line (about 10 min), under UV light at 254 nm and 365 nm after drying the plates.

### β-Glucosidase activity conditions

#### Effect of pH

The β-glucosidase activity was measured on the extracellular fraction resulting from the optimal conditions of β-glucosidase production by the strains studied, including pH (pH5 for FSO1, pH6 for L18 and FSO1/L18), and NaCl concentration (0% of NaCl for all strains). The effect of pH on β-glucosidase activity was measured in 50 mM citrate buffer at pH values of (4, 5, and 6), using 5 mM of p-NPG as substrate. The activity of β-glucosidase was measured according to the method described above. All the experiments were made in triplicate.

#### Effect of NaCl

The effect of NaCl on the β-glucosidase activity of the extracellular fraction of the strains cultures obtained on MRSm was measured in the 50 mM citrate buffer (at pH 5) at 50 °C added with different concentrations of NaCl (0, 2, 4, 6, and 8%, w/v), and using 5 mM of p-NPG as substrate. The activity of the β-glucosidase was measured as described above. All the experiments were made in triplicate.

#### Effect of temperature

The effect of temperature on β-glucosidase activity of the extracellular fraction of strains cultures obtained on MRSm was measured in 50mM citrate buffer (at pH 5), using the p-NPG (5mM) as substrate. The mixture was maintained at temperature values of 6, 15, 25, 35, 45, and 50 °C for 10 min. The residual activity of β-glucosidase was measured as described above. All the experiments were made in triplicate.

#### Statistical analysis

The data obtained from the replicate assays were presented as the means ± standard deviation. The means were compared, with a significant difference at *p* < 0.05, using one-way ANOVA. The results were plotted using Graph Pad Prism, version 8 for Windows, GraphPad Software, San Diego, California, USA.

## Results

### Enzymatic profile and detection of β-glucosidase

The semi-quantitative API-ZYM technique was used to determine the presence and the preliminary quantification of the β-glucosidase enzyme from *Lactobacillus plantarum* FSO1 and *Candida pelliculosa* L18. The results obtained indicate that these strains (FSO1 and L18) produce a wide range of enzymes, with different qualitative and quantitative profiles (Table 1). Among these enzymes, α-glucosidase and β-glucosidase are the most highly detected in both strains. The α-glucosidase score of the API-ZYM scale is 4 (30 nmol) in both strains, while β-glucosidase scores are 3 (20 nmol) and 4 (30 nmol), respectively in FSO1 and L18. Other enzymes, involved in the catabolism of nutrients, are detected at lower contents, such as Naphthol phosphohydrolase, β-galactosidase, alkaline and acid phosphatases, esterase (C4), esterase lipase (C8), lipase (C14), leucine arylamidase, valine arylamidase, cystine arylamidase, trypsin, acid phosphatase, N-acetyl-β-glucosaminidase, α-mannosidase, α-fucosidase.

**Table 1:** Enzymatic activity of oleuropeinolytic strains obtained by API-Zym system

Enzymes	Enzymatic activity (nmol/5 h) level	
	*Lactobacillus plantarum* FSO1	*Candida pelliculosa* L18
Control (no substrate)	0	0
Phosphatase alkaline	2	2
Esterase (C4)	1	2
Esterase lipase (C8)	1	1
Lipase (C14)	1	1
Leucine arylamidase	1	0
Valine arylamidase	1	2
Cystine arylamidase	1	2
Trypsin	1	2
α-Chymotrypsin	0	1
Acid phosphatase	2	2
Naphthol phosphohydrolase	3	2
α-Galactosidase	0	0
β-Galactosidase	1	2
β-Glucuronidase	0	0
α-Glucosidase	4	4
β-Glucosidase	2	4
N-Acetyl-β-glucosaminidase	2	0
α-Mannosidase	0	1
α-Fucosidase	0	1

The scale of the API-Zym test was used for enzymatic quantification, 0 = no enzyme, 1 = 5 nmol, 2 = 10 nmol, 3 = 20 nmol, 4 = 30 nmol and 5 = 40 nmol or more

### Detection of β-glucosidase

**Fig. 1:**
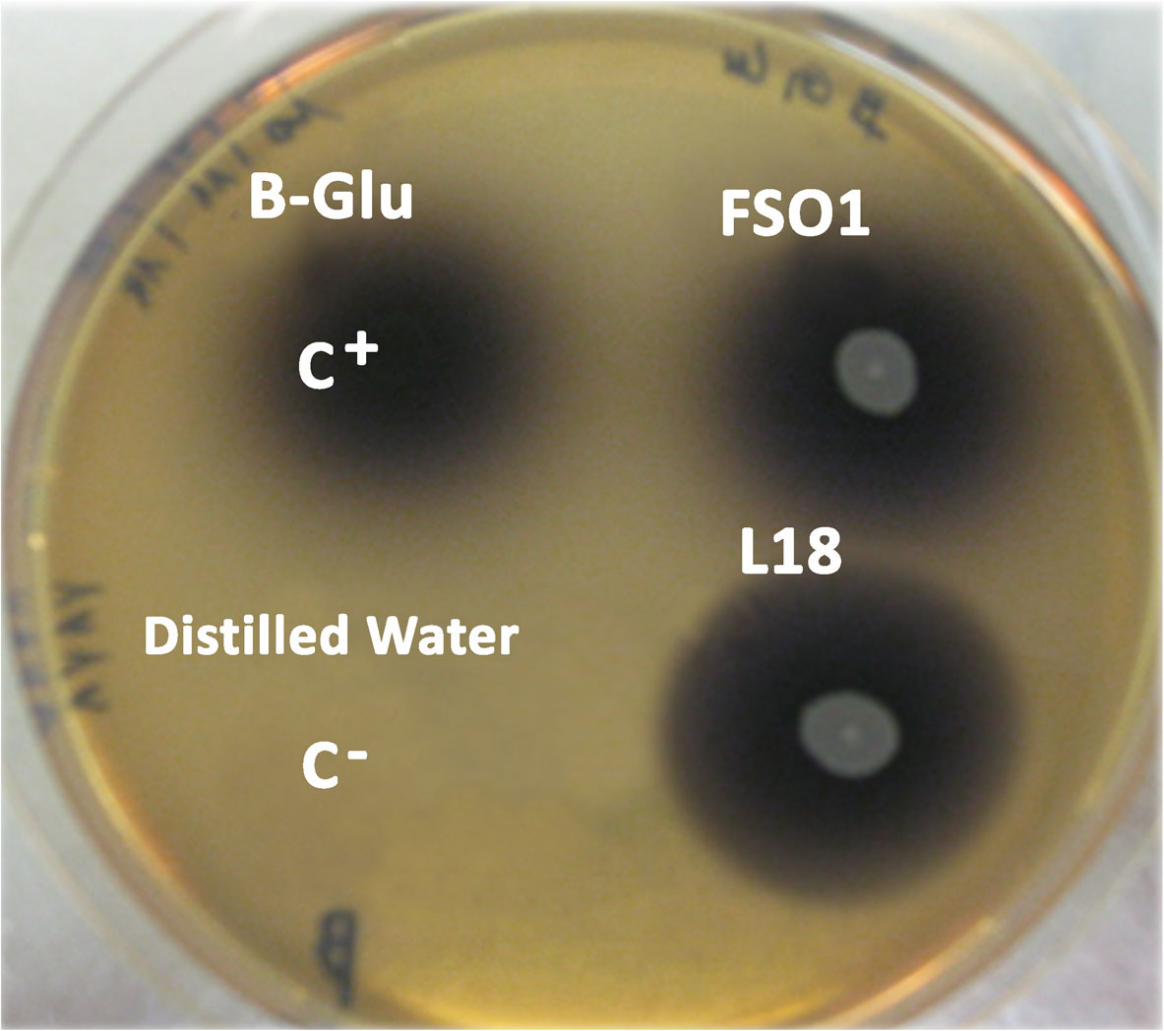
Hydrolysis of esculin obtained on esculin-based medium after 2 days of incubation at 30 °C of *L. plantarum* (FSO1) and *C. pelliculosa* (L18) (legend: C^+^ (B-Glu), commercial enzyme (β-glucosidase EC 3.2.1.21); C^-^: distilled water)

The β-glucosidase activity was detected by the hydrolysis of esculin. Both strains (*L. plantarum* FSO1 and *C. pelliculosa* L18), as well as commercial β-glucosidase (positive control), showed hydrolysis of esculin to form a black precipitate, while no color change was observed with distilled water (negative control) (Fig. 1). The hydrolysis of esculin was determined by the observation of a brown-black color, as a result of the reaction of Fe^3+^ with esculetin, resulting from the hydrolysis of esculin [28].

### Induction and localization of β-glucosidase

**Fig. 2:**
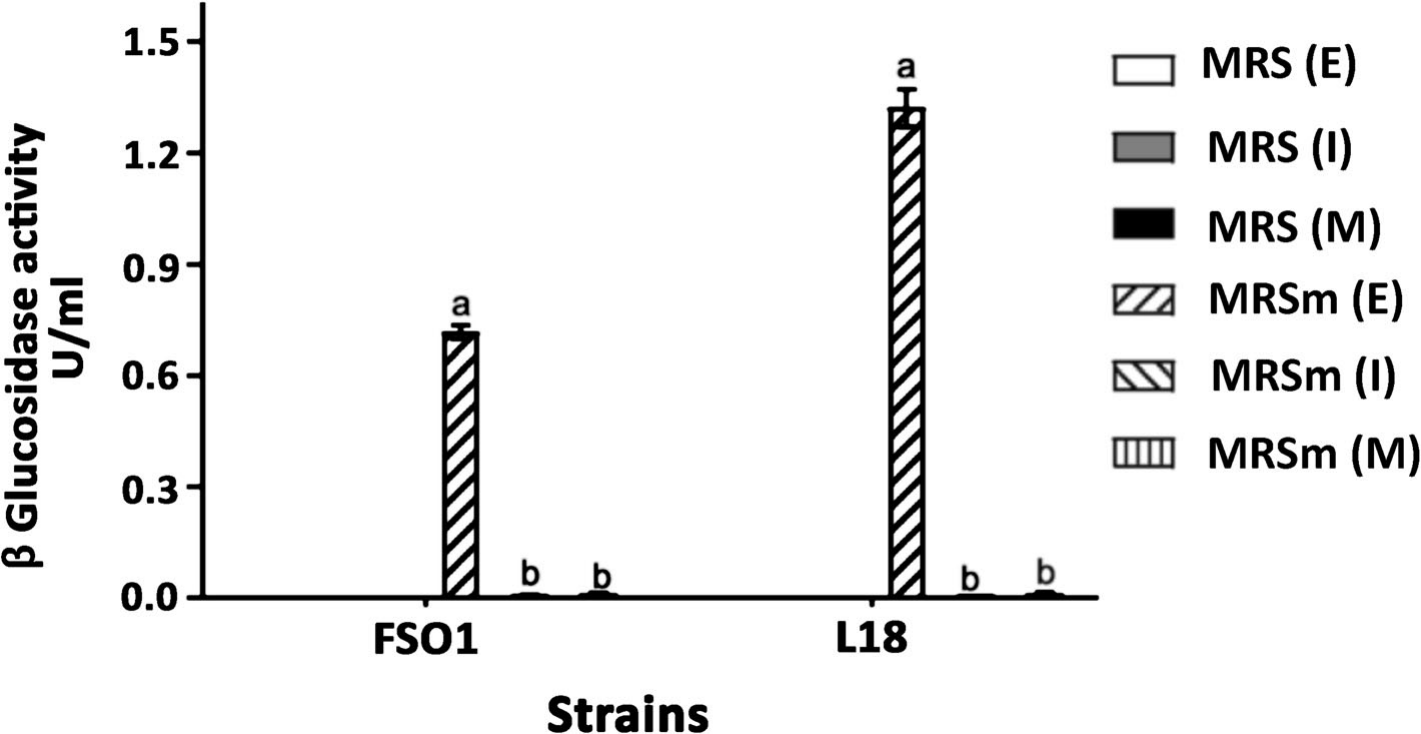
β-Glucosidase activity of *L. plantarum* FSO1 and *C. pelliculosa* L18 obtained after 7 days of culture at 30 °C on MRS and modified MRS (MRSm) containing oleuropein (1%) as a sole carbon source. (Legend: β-glucosidase activity in (*E*): extracellular; (*I*): intracellular and (*M*): parietal fractions)

The results of the induction and localization of β-glucosidase of the studied strains are indicated in Fig. 2. In presence of oleuropein, as a sole carbon source, the β-glucosidase activity values obtained in the extracellular fraction are 1.32 U/mL for *C. pelliculosa* L18 and 0.71 U/mL for *L. plantarum* FSO1, while in absence of oleuropein, a very low activity was detected in all fractions (extracellular: *E*, intracellular: *I*, and cell wall: *M*) of both strains.

#### SDS-PAGE

**Fig. 3:**
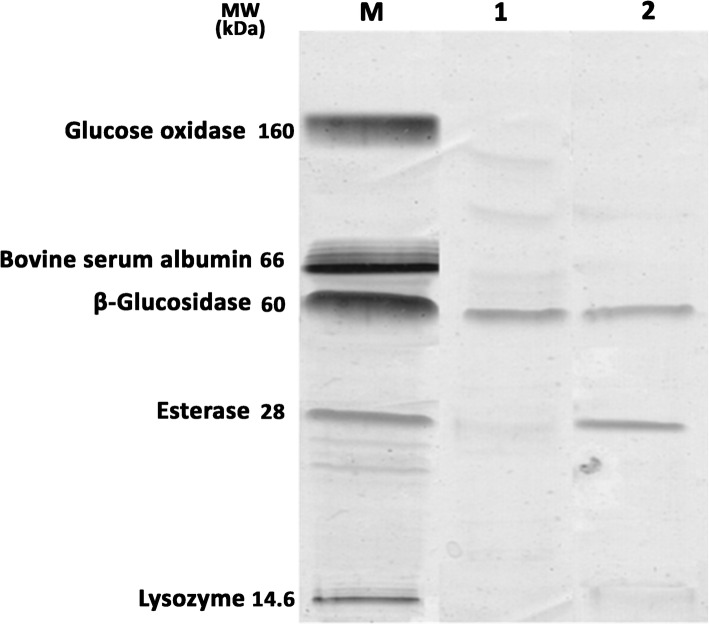
Electrophoretic profile (SDS-PAGE) of proteins extracts of *L. plantarum* FSO1 (lane 1) and *C. pelliculosa* L18 (lane 2) cultures obtained in modified MRS broth (containing OLP 1% as sole carbon source) after 7 days of culture at 30 °C (M: Molecular weight (kDa) of standards)

The results of the enzyme profile of the strains FSO1 and L18, obtained by the SDS-PAGE technique are reported in Fig. 3. Both strains, *L. plantarum* FSO1 and *C. pelliculosa* L18 showed a band of molecular weight of 60 kDa, corresponding to the β-glucosidase enzyme (EC 3.2.1.21) used as standard.

### Confirmation of enzymatic activity by TLC

**Fig. 4:**
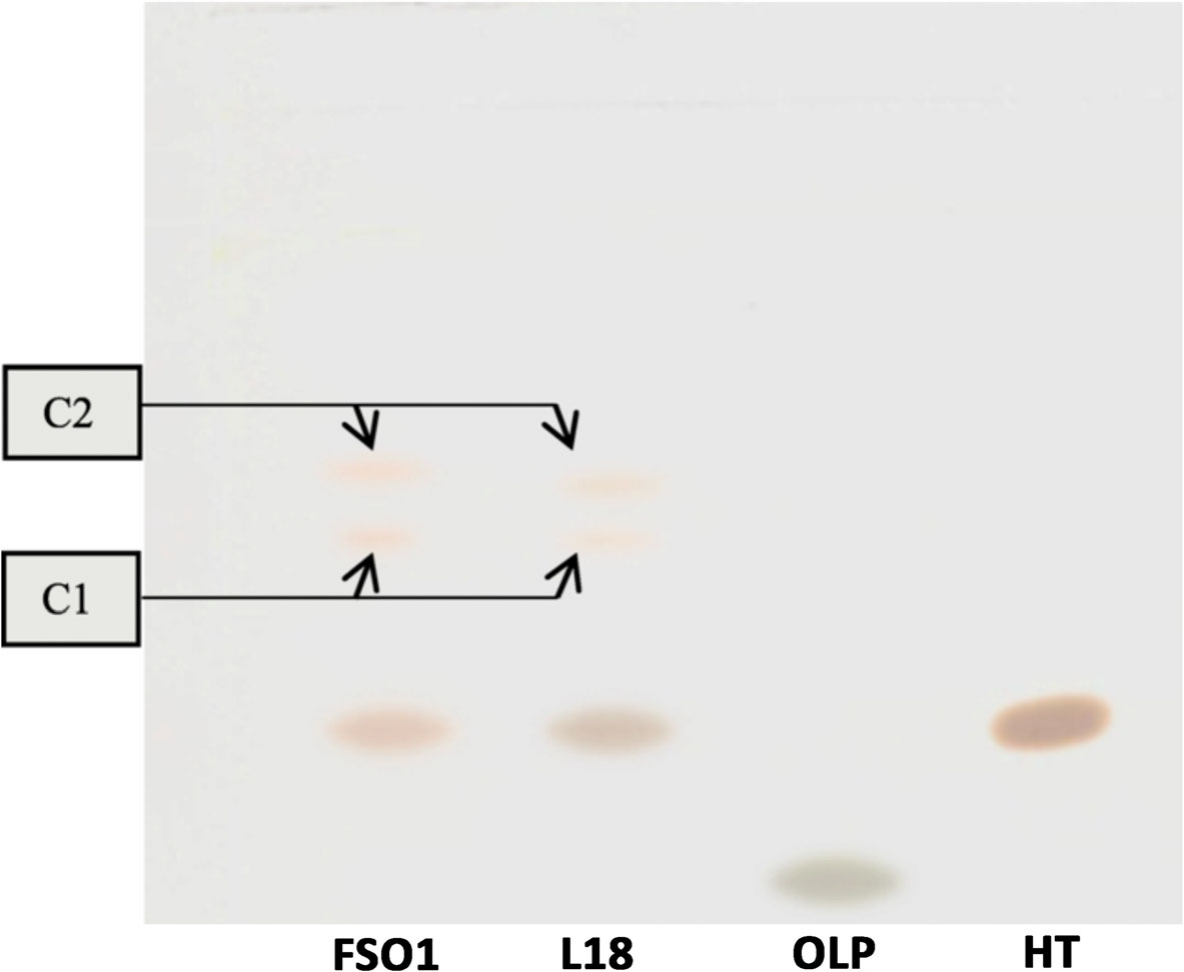
TLC of the methanolic fraction of oleuropeinolytic strains extracts and standards developed by toluene/methanol/acetic acid system (15/5/0.5) and observed under white light. (Legend: FSO1: *L. plantarum* FSO1, L18: *C. pelliculosa* L18, OLP: oleuropein, HT: hydroxytyrosol)

The phenolic compounds extracted from the microbial cultures of (FSO1, L18, and FSO1/L18), obtained after 7 days of incubation at 30 °C on MRSm (containing oleuropein as a sole carbon source), were subjected to thin-layer chromatographic (TLC) separation. The results obtained are reported in Fig. 4. Oleuropein (OLP) and hydroxytyrosol (HT) were used as standards, and the separation system was toluene/methanol/acetic acid (15/5/0.5).

The results indicated the separation of extracted phenolic compounds into several components (spots). The retention factor (Rf) values obtained for the OLP and HT (standards) were 0.16 and 0.37, respectively. All the components obtained showed absorbance at UV 365 nm. The strains FSO1 and L18 demonstrated an absence of OLP spot, and the appearance of HT and 2 other components (C1 and C2), with Rf values of 0.37, 0.48, and 0.54, respectively. These results indicate the production of β-glucosidase and esterase by *L. plantarum* FSO1 and *C. pelliculosa* L18, and it can also be due to the acidification of the medium resulting from the fermentation of glucose released from the degradation of oleuropein by β-glucosidase [29], leading to the elimination of oleuropein and accumulation of hydroxytyrosol.

### Antioxidant activity

**Fig. 5:**
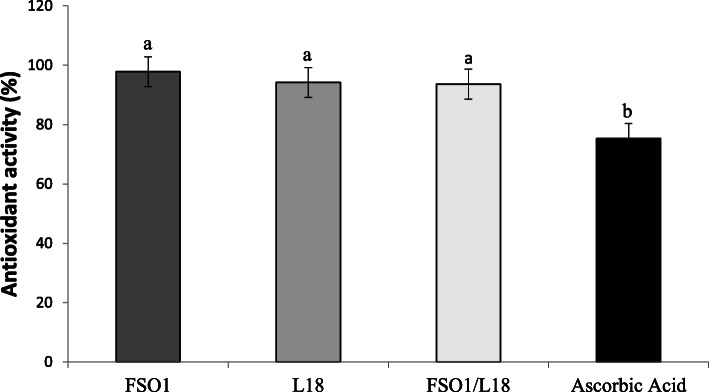
Free radical scavenging activity (%) of cultures of *L. plantarum* FSO1, *C. pelliculosa* L18, and their combination FSO1/L18 obtained after 7 days of incubation at 30 °C in MRSm containing oleuropein as a sole carbon source

The antioxidant activity (AA) values, of the culture of the strains (FSO1, L18, and FSO1/L18) measured by DPPH scavenging activity, are reported in Fig. 5. The values obtained were 97.81% for *L. plantarum* FSO1, 94.17% for *C. pelliculosa* L18, and 93.62% for their combination (FSO1/L18); while ascorbic acid showed an AA value of 75.28%. The AA values of the strains (FSO1, L18, and FSO1/L18) were significantly (*p* < 0.05) higher than the one obtained for ascorbic acid.

### Production of β-glucosidase

#### Effect of incubation time

**Fig. 6:**
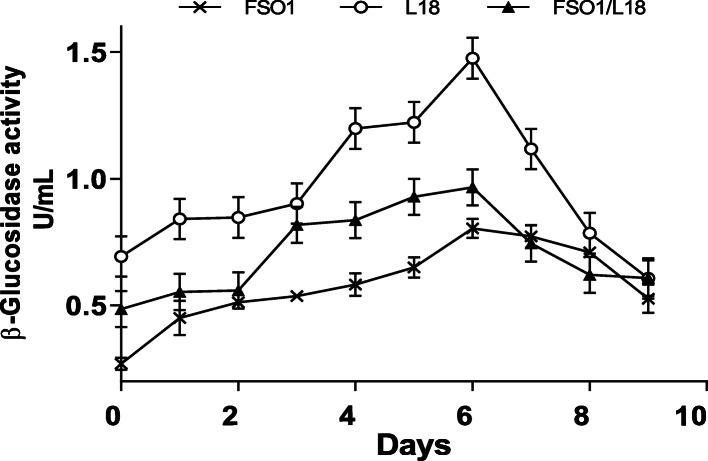
β-Glucosidase activity of pure (FSO1, L18) and combined (FSO1/L18) cultures during 9 days of incubation at 30 °C in modified MRS broth containing oleuropein (1%) as a sole carbon source. (Legend: FSO1: *L. plantarum*, L18: *C. pelliculosa*)

The β-glucosidase (β-Glu) activity values of FSO1 and L18 and their combination (FSO1/L18), obtained during 9 days of culture at 30 °C, are reported in Fig. 6. Both strains and their combination showed an increase in their β-glucosidase activity values until day 7 of culture and decreased thereafter. The maxima of β-glucosidase activity values obtained were 0.77, 1.47, and 0.96 U/mL, respectively for *L. plantarum* FSO1, *C. pelliculosa* L18, and their combination FSO1/L18. The yeast strain L18 displayed a β-glucosidase activity twice that of *L. plantarum* FSO1.

The combination FSO1/L18 showed β-glucosidase activity value significantly (*p* < 0.05) lower than that obtained with L18 alone, and significantly (*p* < 0.05) higher than that obtained with *L. plantarum* FSO1 alone*.* This may be due mainly to the decrease of pH to a lower value than the optimum pH of β-glucosidase, due to organic acids production by *L. plantarum* FSO1.

**Fig. 7:**
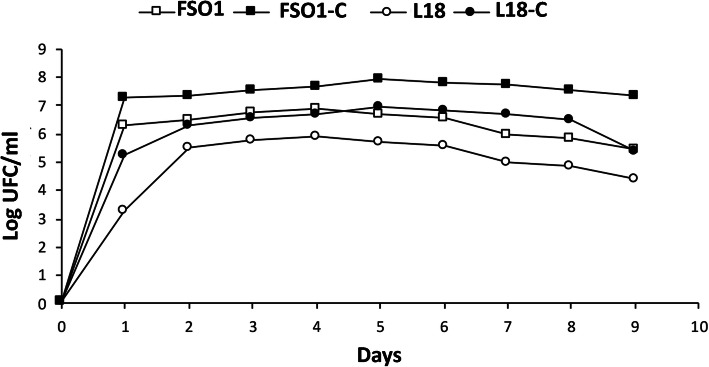
Evolution of biomass growth of FSO1 and L18 in pure cultures and combined culture (FSO1-C, L18-C) in modified MRS medium (Legend: FSO1: *L. plantarum*, L18: *C. pelliculosa*, FSO1-C: FSO1 in FSO1/L18, L18-C: L18 in FSO1/L18)

The biomass values of FSO1 and L18 obtained in their pure or mixed culture are reported in Fig. 7. For each strain (FSO1 or L18), the biomass values obtained in pure culture are lower than those obtained in mixed cultures.

#### Effect of pH on β-glucosidase production

**Fig. 8:**
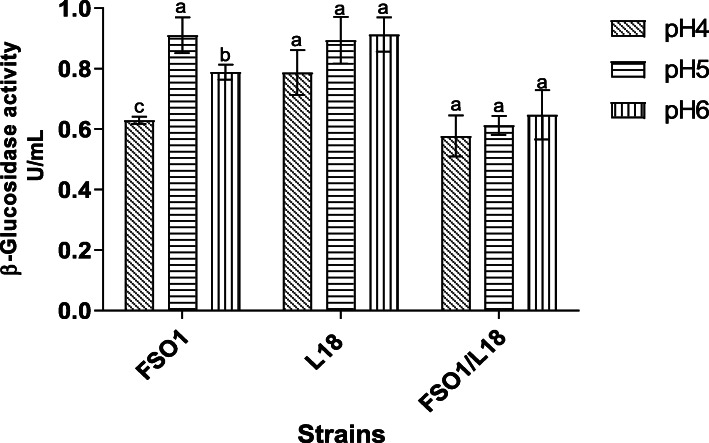
β-Glucosidase activity of strains (FSO1, L18, and FSO1/L18) cultures obtained after 7 days of incubation at 30 °C in MRSm adjusted to different initial pH (4, 5 and 6). (Legend: MRSm: MRS containing oleuropein (1%) as a sole carbon source, FSO1: *L. plantarum* FSO1, L18: *C. pelliculosa* L18, FSO1/L18: combination of FSO1 and L18)

The β-glucosidase activity values obtained for the strains (FSO1, L18, and FSO1/L18) after 7 days of culture at 30 °C on MRSm (MRS medium containing oleuropein (1%) as sole carbon source) adjusted to different initial pH (4, 5, and 6) are displayed in Fig. 8. *L. plantarum* FSO1 showed optimum production of β-glucosidase (0.91 U/mL) at pH 5, while the values obtained at pH 4 (0.63 U/mL) and pH 6 (0.79 U/mL) were significantly (*p* < 0.05) lower.

#### Effect of NaCl on β-glucosidase production

**Fig. 9:**
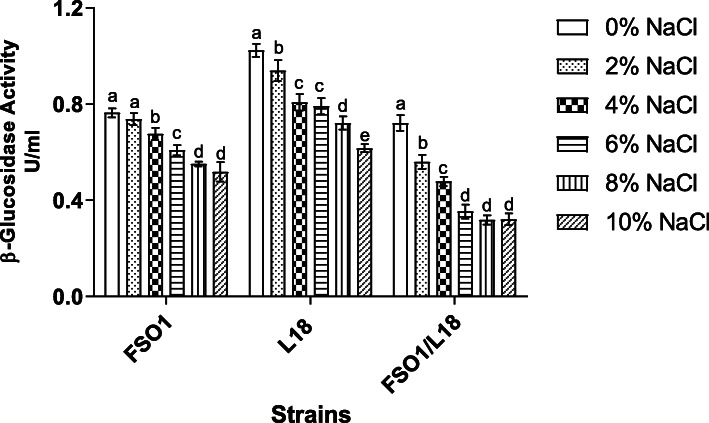
β-Glucosidase activity produced by strains FSO1, L18 and FSO1/L18 after 7 days of culture at 30 °C in MRSm adjusted to different initial NaCl concentrations (Legend: MRSm: MRS containing oleuropein (1%) as a sole carbon source, FSO1: *L. plantarum* FSO1, L18: *C. pelliculosa* L18, FSO1/L18: combination of FSO1 and L18)

The effect of NaCl (0, 2, 4, 6, 8, and 10%, w/v) on the production of β-glucosidase, by the strains (FSO1, L18, and FSO1/L18) on MRSm medium (containing oleuropein 1% as sole carbon source), is reported in Fig. 9. The maximum β-glucosidase activity was obtained in the absence of NaCl (0%), and the values obtained were 0.76 U/mL, 1.02 U/mL, and 0.72 U/mL, respectively for *L. plantarum* FSO1, *C. pelliculosa* L18, and their combination FSO1/L18. The production of β-glucosidase decreased, with the increase of NaCl to reach minimum activity values of 0.51 U/mL, 0.61 U/mL, and 0.32 U/mL, obtained at 10% NaCl for FSO1, L18, and FSO1/L18, respectively.

### Activity of β-glucosidase

#### Effect of pH on β-glucosidase activity

**Fig. 10:**
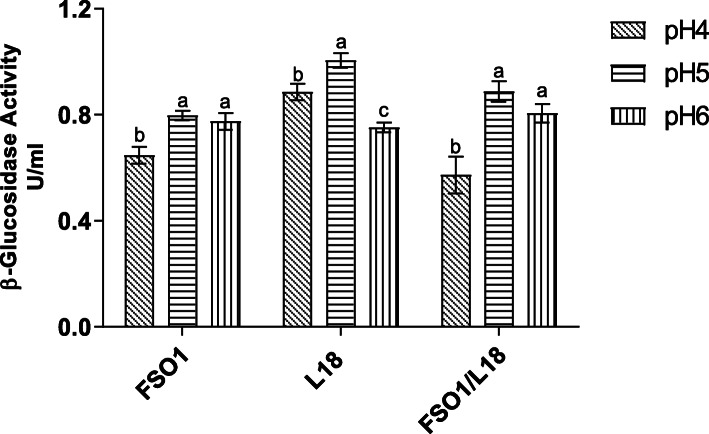
Residual activity of β-glucosidase after 7 days of cultures at 30 °C for pure (FSO1, L18) and combined (FSO1/L18) cultures in modified MRS broth containing oleuropein (1%) as a sole carbon source at different final pH. (Legend: FSO1: *L. plantarum*, L18: *C. pelliculosa*).

The effect of pH on the β-glucosidase activity of the strains FSO1, L18, and FSO1/L18 is reported in Fig. 10. The optimum β-glucosidase residual activity value was obtained at pH 5 for all strains, with activity values of 0.79 U/mL for *L. plantarum* FSO1, 1 U/mL for *C. pelliculosa* L18, and 0.88 U/mL for their combination (FSO1/L18). The optimum residual activity values of L18 and FSO/L18 are higher than that of *L. plantarum* FSO1.

#### Effect of NaCl on β-glucosidase activity

**Fig. 11:**
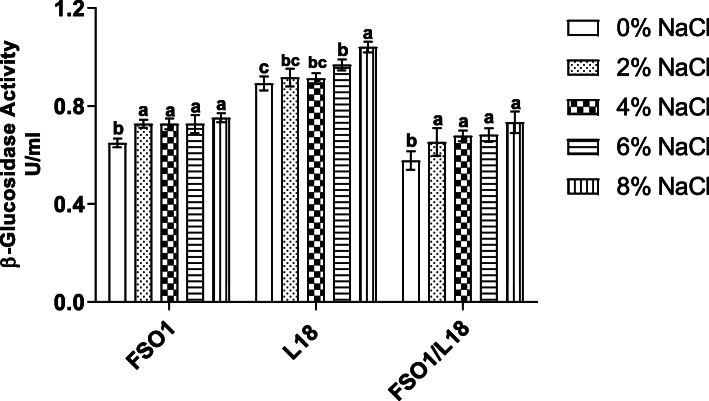
Residual activity of β-glucosidase after 7 days of cultures at 30 °C for pure (FSO1, L18) and combined (FSO1/L18) cultures in modified MRS broth containing oleuropein (1%) as a sole carbon source at different final NaCl concentrations. (Legend: FSO1: *L. plantarum*, L18: *C. pelliculosa*)

The effect of NaCl on the β-glucosidase activity of the strains (FSO1, L18, and FSO1/L18) is reported in Fig. 11. The β-glucosidase activity values of all strains increased slightly from 0 to 4%, and highly from 4 to 8%. The maximum of β-glucosidase residual activity values was obtained at 8% (w/v) NaCl, with activity values of 0.75 U/ mL for *L. plantarum* FSO1, 1.04 U /mL for *C. pelliculosa* L18, and 0.73 U/mL for their combination (FSO1/L18). These residual activity values are significantly different (*p* < 0.05) between pure strains FSO1, L18 and FSO1/L18, and indicate the highest activity of *C. pelliculosa* L18, followed by the combination FSO1/L18 and finally by *L. plantarum* FSO1.

#### Effect of temperature on β-glucosidase activity

**Fig. 12:**
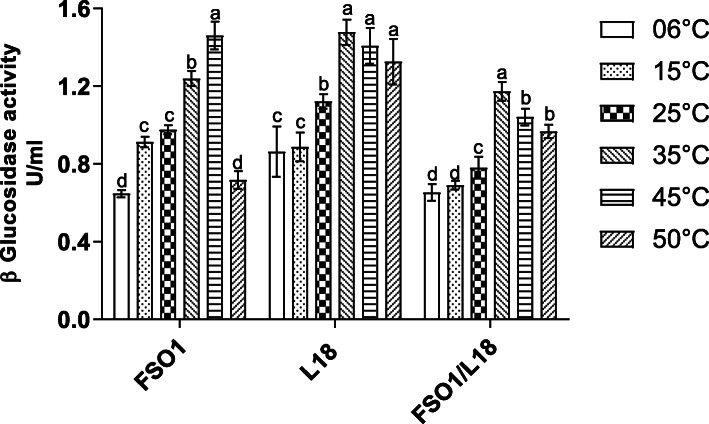
Residual activity of β-glucosidase after 7 days of cultures at 30 °C for pure (FSO1, L18) and combined (FSO1/L18) cultures in modified MRS broth containing oleuropein (1%) as a sole carbon source at different final temperatures. (Legend: FSO1: *L. plantarum*, L18: *C. pelliculosa*)

The effect of temperature on the activity of β-glucosidase of the strains (FSO1, L18, and FSO1/L18) is reported in Fig. 12. The optimum β-glucosidase activity of L18 and FSO1/L18 was obtained at 35 °C, with maximum activity values of 1.48 and 1.17 U/mL, respectively; While for FSO1, the optimum was obtained at 45 °C, with a residual activity value of 1.46 U/mL.

## Discussion

Both strains, *L. plantarum* FSO1 and *C. pelliculosa* L18 showed hydrolysis of esculin by producing the β-glucosidase enzyme. Previous studies reported the β-glucosidase activity in lactic acid bacterial strains [15, 30–33], and yeast strains [34, 35], isolated from olive brine.

High levels of β-glucosidase activity were detected in both strains (*L. plantarum* FSO1 and *C. pelliculosa* L18). This may be due to their natural selection in the spontaneous fermentation process of non-debittered green olive brine. The traditional fermentation process of non-debittered green olive is characterized by high contents of phenolic compounds (oleuropein) and NaCl (0–12%) and lower nutrients contents [17, 36]. The microbial strains, involved in this fermentation process, should have an important and wide enzymatic profile, mainly β-glucosidase, to overcome the stress factors of this extreme environment. The β-glucosidase activity is frequent in lactic acid bacterial strains isolated from the traditional non-alkali-treated green olive fermentation process [15].

The results indicate that the production of β-glucosidase by these strains is extracellular and induced by oleuropein. This result is in agreement with that found by Sestelo, Poza [10]. Other studies reported, however, high β-glucosidase activity in intracellular fractions of *Lactobacillus* and yeast [13, 37, 38]. The MW (60 kD) of the extracellular β-glucosidase we obtained in these strains (FSO1 and L18), is comparable to that reported for extracellular β-glucosidase [39, 40], but lower than that reported for intracellular β-glucosidase [37, 38]. The esterase enzyme of MW 28 kDa, obtained in *C. pelliculosa* L18, is comparable to that obtained by Esteban-Torres, Reveron [41]. The esterase was not detected in *L. plantarum* FSO1, which may be due to its lower content.

The degradation of OLP by FSO1 and L18 strains led to the appearance of HT and 2 other components (C1 and C2), revealed by TLC. These compounds (C1 and C2) may correspond to intermediate phenolic compounds, other than HT, such as hydroxytyrosol glucoside, tyrosol, 3,4-dihydroxy phenylacetic acid, or elenolic acid [42]. HT was reported as the final product of oleuropein biodegradation [1]. It was demonstrated that the biodegradation of oleuropein by β-glucosidase results in glucose and aglycone [3, 39], and thereby, the aglycone is degraded by esterase to simpler phenolic compounds, namely hydroxytyrosol and elenolic acid [1, 4].

The higher AA values, obtained with culture extracts of the strains (FSO1, L18, and FSO1/L18), may be due to oleuropein and its hydrolysis products, particularly hydroxytyrosol, which is highly desired in foods [43, 44]. The culture extracts of the strains demonstrated the biodegradation of oleuropein and accumulation of hydroxytyrosol and other phenolic compounds, leading to antioxidant activity values significantly higher than that of ascorbic acid.

The enhancement of biomass growth, observed in a mixed culture of *L. plantarum* FSO1 and *C. pelliculosa* L18, may be due to mutual metabolic nutrients exchange between these strains. Metabolic cross-feeding interactions were demonstrated in microbial communities of yeasts and *Lactobacillus*, particularly in the nutrient-limited environment [45, 46].

The lowest β-glucosidase activity value observed at pH 4, may be due to the reduction of biomass growth and to pH change far from the optimum pH of the enzyme. The reduction of biomass growth with a decrease of pH was demonstrated in *Lactobacillus* strains isolated from olive brine [11]. It was reported that the pH range affects the production of β-glucosidase in *Lactobacillus plantarum* [47]. For *C. pelliculosa* L18, the β-glucosidase activity value increased, not significantly (*p* < 0.05), with an increase of pH to a maximum activity value of 0.91 U/mL obtained at pH 6. These results indicate the wide pH range of the production of β-glucosidase in *C. pelliculosa* L18. The optimum pH of β-glucosidase production reported by other researchers for yeasts ranged between 5 and 6.5 [48, 49].

The combination FSO1/L18 produced β-glucosidase activity values lower than those obtained with each strain alone. The β-glucosidase activity value increased, not significantly (*p* < 0.05), with an increase of pH to reach the maximum activity of 0.64 U/mL obtained at pH 6. These findings may lead us to conclude that the production of β-glucosidase is dependent on the initial pH for *L. plantarum* FSO1, but not for *C. pelliculosa* L18*.*

The production of β-glucosidase is limited with the increase in NaCl concentration. This result confirms the hypothesis of Gouripur and Kaliwal [47]. The reduction of β-glucosidase production may be due to the inhibition of biomass growth caused by the increase of the osmotic pressure of NaCl.

The hydrolytic activity of the enzyme has optimum pH of 5, which is similar to that of many β-glucosidases isolated from the *Plumeria obtusa* (white frangipani) flower [40, 50], and lower than the optimum pH (7.5) obtained for *Lactobacillus* isolated from dairy products [51]. The enzyme retained 75% relative activity in the pH range of 4.5–6.5 [40].

The β-glucosidase activity increased with the increase of NaCl concentration, particularly from 4 to 8%. This result is in agreement with the previous work of Restuccia, Muccilli [52]. This stimulation of β-glucosidase by NaCl may be due to the increase of the ionization of the reactional solution of the enzyme [53–55].

The optimum temperature (45 °C) is higher than that obtained (30 °C) for *L. casei and L. rhamnosus* isolated from dairy products by Yuksekdag, Cinar Acar [51]. The β-glucosidase activity value of FSO1 decreased drastically at 50 °C. This result is in agreement with the previous result of Restuccia, Muccilli [52].

## Conclusions

The present study was focused on the characterization of β-glucosidase of the strains *L. plantarum* FSO1 and *C. pelliculosa* L18, isolated from traditional fermented green olive. The strains studied showed extracellular and induced production of β-glucosidase, with an MW of 60 kD. The production of β-glucosidase was higher in *C. pelliculosa* L18 than *L. plantarum* FSO1, while intermediate activity values were obtained with their combination (FSO1/L18). The maximum activity value of all strains was obtained after 6 days of culture and decreased thereafter. The production of β-glucosidase was optimal at pH 5 and 30 °C for *L. plantarum* and pH 6 and 30 °C for *C. pelliculosa* L18. The activity of the enzyme was optimal at pH 5 and 45 °C for *L. plantarum* FSO1, and at pH 5 and 35 °C for *C. pelliculosa* L18. The increase of NaCl concentration reduced the production of β-glucosidase by the strains, while their enzyme activity increased. The strains *L. plantarum* FSO1 and *C. pelliculosa* L18 and/or their enzyme extract showed promising traits for their application in the biological processing of table olives.

## Data Availability

All data generated or analyzed during this study are included in this article.

## Abbreviations

%: Percentage
AA: antioxidant activity
ANOVA: Analysis of variance
C: *Candida*
°C: Degree Celsius
CNRST: Centre National pour la Recherche Scientifique et Technique
DPPH: 2;2-Diphenyl-1-picrylhydrazyl
g: Gram
HT: Hydroxytyrosol
kDa: Kilodalton
L: *Lactobacillus*
l: Liter
ml: Milliliter
M: Molar
mM: Millimolar
MRS: De Man Rogosa and Sharp
MRSm: Modified De Man Rogosa and Sharp
NaCl: Sodium chloride
OLP: Oleuropein
pH: Hydrogen potential
p-NP: Para-nitrophenol
p-NPG: Para-nitrophenyl β-d-glucopyranoside
Rf: Frontal ratio
SDS: Sodium dodecyl sulfate
SDS-PAGE: Sodium dodecyl sulfate-polyacrylamide gel electrophoresis
TLC: Thin-layer chromatography
U: International unit
UV: Ultraviolet
μg: Microgram
μl: Microliter
v/v: Volume per volume
w/v: Weight per volume
